# Advancements in Dual-Load Antibody–Drug Conjugates and Challenges with Quality Analysis

**DOI:** 10.3390/ph19060860

**Published:** 2026-05-29

**Authors:** Xiaojuan Yu, Xiao Ke, Yao Tang, Tao Tang, Yongbo Ni, Luyun Guo, Yongfei Cui, Yuting Mei, Gangling Xu, Gang Wu, Yalan Yang, Maoqin Duan, Jialiang Du, Meng Li, Jiao Tang, Shijun Yin, Jiali Zuo, Yanhua Xu, Yonghao Zhao, Yan Li, Chuanfei Yu

**Affiliations:** 1 National Institutes for Food and Drug Control, NHC Key Laboratory of Research on Quality and Standardization of Biotech Products, NMPA Key Laboratory for Quality Research and Evaluation of Biological Products, State Key Laboratory of Drug Regulatory Science, Beijing 102629, China; 2 Chengdu Kanghong Pharmaceutical Group Co., Ltd., Chengdu 610037, China; 3 Sichuan Institute for Drug Control, NMPA Center for Innovation and Research in Regulatory Science, Chengdu 611731, China

**Keywords:** dual-payload antibody–drug conjugates, quality control, liquid chromatography–mass spectrometry, drug-to-antibody ratio, analytical methods

## Abstract

Antibody–drug conjugates (ADCs) are a pivotal technology for precision cancer therapy, harnessing the synergistic effects of antibody targeting and toxin delivery. However, traditional ADCs encounter limitations in efficacy that stem from tumor resistance, heterogeneity, and intense target competition. Dual-payload ADCs (DP-ADCs) represent a promising solution to these challenges, as they leverage dual mechanisms of action that mitigate acquired drug resistance and enhance adaptability to tumor heterogeneity. The complex structure of DP-ADCs presents substantial quality control hurdles. In this manuscript, we review the current payload selection and conjugation strategies of DP-ADCs and examine recent advances in quality control research. Specifically, we analyze the analytical challenges related to the quantification of free toxins, the determination of the total antibody content, and the characterization of the drug-to-antibody ratio and its distribution. Ultimately, the aim of this work is to provide valuable guidance for future DP-ADC quality control analyses to facilitate their clinical translation and application.

## 1. Introduction

Antibody–drug conjugates (ADCs) are highly targeted therapeutics comprising antibodies, a linker, and payloads. They are designed to combine the high specificity of antibodies with the functional activity of payloads. This approach enables highly selective targeting and the efficient killing of tumor cells [1]. ADCs typically engage with target antigens on tumor cells, are internalized via endocytosis, and subsequently release their payloads through enzymatic hydrolysis or non-enzymatic mechanisms at the linker site, ultimately inducing tumor cell apoptosis [2]. Compared with traditional anticancer drugs, ADCs offer a wider therapeutic window and a higher therapeutic index, markedly improving treatment efficacy and safety [3]. Currently, 23 ADCs have been approved worldwide for hematologic malignancies and solid tumors, with hundreds more under clinical evaluation, reflecting substantial market potential [4].

Despite these advances, the clinical discontinuation of over 150 ADC candidates underscores significant developmental challenges. Dose-dependent toxicity can impose a limit on the desired therapeutic dose based on the maximum tolerated dose (MTD) [5]. Moreover, the emergence of drug-resistant cancer cell populations that develop under therapeutic pressure remains a major obstacle for traditional ADC therapies [6]. In other words, the administration of a single therapeutic drug exerts a selective pressure that can promote the survival and proliferation of drug-insensitive tumor cells, leading to acquired drug resistance [7]. Consequently, many chemotherapy regimens employ combination therapies with complementary mechanisms of action to reduce resistance and enhance efficacy to achieve positive outcomes in anti-tumor treatments [8,9]. To maximize the efficacy of combination therapies and overcome and prevent treatment resistance, ADC research has been drawing inspiration from and shifting toward the development of dual-drug or multi-drug delivery systems [10].

**Figure 1:**
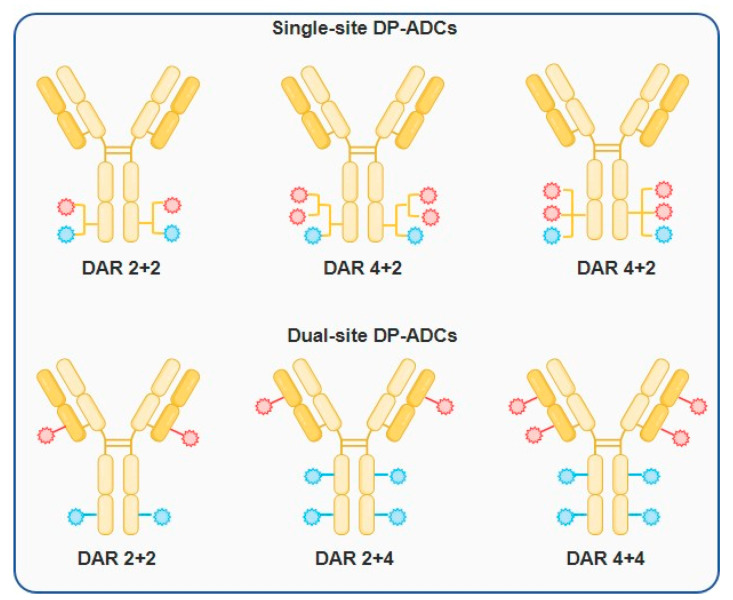
Multiple designs with different DAR and payload ratios for DP-ADCs (constructed and processed with Adobe Illustrator 29.8.4, Blender 4.3.0 and Microsoft PowerPoint 2010) [12,13].

Due to their promising therapeutic potential, dual-payload ADCs (DP-ADCs) are increasingly being recognized as a key research area for ADC development and are advancing into the preclinical and clinical trial phases [11]. DP-ADCs are more than a simple combination of two payloads: their design requires a sophisticated strategy that includes careful payload selection and coordinated optimization of antibodies and linkers. Recent publications show multiple designs with different DAR and payload ratios for DP-ADCs [12,13], as shown in Figure 1. Additionally, the complex structure of DP-ADCs presents unprecedented quality control challenges. These include analytical challenges related to quantifying free toxins, determining the total antibody content, and characterizing the drug-to-antibody ratio (DAR) and its distribution [14]. Consequently, the development and implementation of advanced, high-precision analytical methodologies are urgently needed to guarantee the safety, efficacy, and reproducibility of these therapeutics [15]. Developing thorough and comprehensive DP-ADC quality control assessments is a crucial step for clinical translation to ensure their potential to benefit patients with cancer can be realized, as shown in Table 1.

Due to their unique therapeutic mechanism, DP-ADCs pose broad application prospects for tumor treatment. However, the complexity of DP-ADCs introduces numerous challenges for quality analyses that necessitate further research and analytical refinement. We explore these challenges in this review, beginning with a critical evaluation of the current payload selection strategies, the introduction of potential payload candidates, and the coupling strategy for DP-ADCs. We then comprehensively examine the current landscape of quality analyses that have been applied to DP-ADCs, focusing on the unique challenges inherent to their characterization and quality control processes. The aim of this review is to enhance the consistency and efficacy of DP-ADC quality control assessment and facilitate further advancements in DP-ADC development.

## 2. Application of Payload and Coupling Technology in DP-ADCs

**Figure 2:**
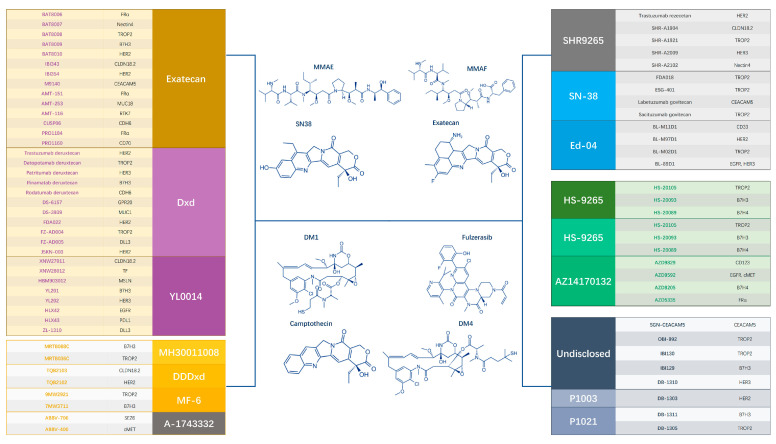
The structures of common payloads used in ADCs and currently representative in-development ADC products. MMAE, MMAF, SN38 and DM1 have been used as payloads in DP-ADCs, as shown in Table 2 (constructed and processed with Kingdraw 3.6.9 and Microsoft PowerPoint 2010).

The selection of an appropriate payload is a critical design consideration for DP-ADCs as it influences their mechanism of action. DP-ADCs often require two distinct conjugation methods, such as interchain cysteine conjugation, engineered cysteine conjugation, engineered non-natural amino acid incorporation, and enzyme-catalyzed site-specific conjugation. Therefore, in this section, we introduce types of payloads and conjugation technologies that are currently utilized in the reported DP-ADCs. Figure 2 and Table 2 summarizes the current landscape of these DP-ADCs [13,18,19,20,21,22,23,24,25,26,27,28,29,30,31,32,33,34,35,36,37,38,39,40].

### 2.1. Auristatins

Monomethylauristatin E (MMAE) and monomethylauristatin F (MMAF), derivatives of dolastatin, are prominent payloads in ADCs due to their potent microtubule-inhibiting activity [34]. Their molecular designs preserve a robust antitumor efficacy while possessing favorable physicochemical properties for ADC development. Both compounds bind to the β-subunit of tubulin, disrupting microtubule dynamics, arresting cell mitosis, and ultimately inducing apoptosis that exhibits a 100- to 1000-fold greater potency than conventional chemotherapeutic agents such as doxorubicin [41]. The high membrane permeability of MMAE facilitates a bystander effect that targets antigen-negative neighboring cells and mitigates the challenges posed by tumor heterogeneity [42,43]. Conversely, MMAF’s C-terminal is a charged phenylalanine residue. This distinguishes it from MMAE, which introduces a negative charge at a physiological pH, restricting cell membrane penetration and minimizing off-target cytotoxicity, thereby selectively targeting antigen-positive cells and reducing systemic toxicity in heterogeneous tumors [44]. Both compounds demonstrate excellent metabolic stability in plasma and hepatic lysate extracts and against cathepsin B. This ensures circulatory integrity and targeted drug release within tumors. During ADC design, MMAE is commonly linked to antibodies via protease-sensitive linkers, such as the valine-citrulline (VC) linker. This promotes efficient intracellular cleavage. MMAF, however, frequently utilizes non-cleavable linkers to minimize extracellular release and optimize the balance between efficacy and safety. Several MMAE or MMAF-based ADCs [45,46,47,48,49], including brentuximab vedotin (Adcetris^®^), polatuzumab vedotin (Polivy^®^), enfortumab vedotin (Padcev^®^), belantamab mafodotin (Blenrep^®^), and tislelizumab (Tivdak^®^), have received regulatory approval for indications such as lymphoma, urothelial carcinoma, and cervical cancer, thereby underscoring their clinical utility as ADC payloads.

In 2017, Levengood et al. [18] reported the first DP-ADC, which conjugated MMAE and MMAF on a short peptide linker containing ortho-protected cysteine residues with a DAR of 16 (8 + 8). To synthesize the ADC, interchain disulfides in the native antibody were first reduced using tris(2-carboxyethyl)phosphine. The resulting free thiols were then added to maleimide side chains to couple the dual-Cys linker. This allowed for the mounting of eight linkers on the antibody, enabling the attachment of sixteen payloads. Subsequent sequential unmasking of the cysteine residues facilitated conjugation of two distinct drugs. The resulting DP-ADCs exhibited potent antitumor activity while overcoming tumor cell resistance through the addition of complementary payloads.

To address challenges associated with ADC coupling uniformity and to obtain different DAR values, Yamazaki et al. [13] developed a DP-ADC that used click chemistry to achieve highly homogeneous conjugates with flexible DAR configurations (2 + 2, 4 + 2, and 2 + 4). A branched ADC linker enabled the site-specific and quantitative installation of two identical payload molecules onto a single antibody via orthogonal strain-promoted azide-dibenzocyclooctyne (DBCO) cycloaddition. To facilitate the assembly of dual-drug ADCs that use this technology, a novel branched linker incorporating azide and methyltetrazine groups as orthogonal click handles was prepared, leveraging methyltetrazine-trans-cyclooctene chemistry as a secondary click chemistry pair. In vitro studies demonstrated that these DP-ADCs retained potent antitumor activity against the HER2-low, chemotherapy-resistant JIMT-1 breast cancer cell line. Moreover, in vivo xenograft models with HER2 heterogeneity and resistance revealed that DP-ADCs possessed significantly improved antitumor efficacy compared with single-agent ADCs or combined treatments with individual payloads.

Similarly, Tang et al. [19] recently developed a simple and efficient one-pot method for the simultaneous construction of dual-site-specific ADCs that enabled precise control over the DAR (2 + 2) at both the N-glycosylation sites and the K248 residue. These ADCs demonstrated excellent homogeneity and buffer stability. In vitro studies revealed significant cytotoxic activity against HER2-positive cell lines, particularly when utilizing a dual-toxin ADC with a DAR of 4. In vivo experiments using the NCI-N87 model indicated that the MMAF-based ADCs exhibited limited antitumor activity, while those loaded with MMAE displayed optimal therapeutic efficacy.

### 2.2. Maytansinoids

Maytansine is another important class of microtubule-disrupting agents frequently employed as ADC payloads. It was originally isolated from the bark of Maytenus ovatus (an African shrub). Maytansine binds to the β1 subunit of tubulin, thereby inhibiting tubulin polymerization [50]. In vitro studies have shown that maytansine effectively inhibits tumor cell proliferation and differentiation at picomolar concentrations [51]. Its excellent chemical stability and water solubility make it a promising drug payload for ADCs. Native maytansine lacks readily modifiable reactive groups; hence, direct conjugation to antibodies is challenging. To address this limitation, researchers have developed structural modifications. For example, DM1 was produced by replacing the N-acetyl group on the C3 side chain of maytansine, where the N-acetyl group is substituted with a 3-(methylthio) propionaldehyde group. Subsequent methylation of the disulfide bond in DM1 resulted in DM4 [52,53]. The disulfide bonds in DM1 and DM4 allow them to be conjugated to linkers, thereby facilitating their attachment to antibodies. Notably, Genentech successfully launched T-DM1 in 2013, the first clinically approved ADC that uses DM1 as its payload, for the treatment of HER2-positive advanced metastatic breast cancer.

To enhance the anti-tumor activity of trastuzumab (Tmab), Candice et al. [20] developed a DM1 and MMAE DP-ADC. First, Tmab, a humanized anti-HER2 monoclonal antibody, was coupled with MMAE using a cleavable VC linker. This generated Tmab-VcMMAE ADC. Subsequently, they incorporated the mitotic inhibitor, DM1, via a non-cleavable succinimidyl4-(N-maleimidomethyl) cyclohexane-1-carboxylate (SMCC) linker to create the dual-conjugate Tmab-VcMMAE-SMCC-DM1. In vitro studies demonstrated that this DP-ADC significantly reduced GR50 values in breast and colon cancer cell lines, indicating enhanced cytotoxic effects.

### 2.3. Camptothecin

Unlike microtubule inhibitors, topoisomerase inhibitors are frequently employed as payloads in ADCs. Camptothecin is an alkaloid derived from the bark of Camptotheca trees. It inhibits DNA replication and transcription by targeting topoisomerase I and ultimately induces tumor cell death. However, its clinical application has historically been limited due to factors including hematologic and gastrointestinal toxicity, poor solubility, low oral bioavailability, rapid in vivo clearance, and lactone ring inactivation. Renewed interest in camptothecin-based drugs has emerged with the successful approval of irinotecan (for metastatic colorectal cancer) and topotecan (for ovarian cancer). Currently, many camptothecin derivatives are undergoing clinical investigations, as shown in Figure 2 [54]. The recent success of two FDA-approved ADCs that employ camptothecin analogs as payloads (namely, Tmab deruxtecan and sacituzumab govitecan, which employ droncitane [DXd] and 7-ethyl-10-hydroxycamptothecin [SN-38], respectively) has significantly amplified research and development efforts focused on this class of compounds.

Wilski et al. [21] reported the synthesis of a novel DP-ADC that uses a unique trifunctional linker. SN38, a topoisomerase inhibitor, was coupled to a cleavable PEG8-VC-PABC linker via copper-catalyzed azide-alkyne cycloaddition, while MMAF, a microtubule polymerization inhibitor, was coupled to a non-cleavable PEG4-aminooxy linker. The tri-functional linker with MMAF and SN38 was conjugated to a bi-specific antibody against EGFR and cMET using maleimide conjugation to produce a targeting DP-ADC (412a-MMAF + SN38). In vitro and in vivo evaluations revealed that 412a-MMAF + SN38 demonstrated superior inhibitory activity against cell lines resistant to ADC-based therapies. Animal studies have confirmed that this DP-ADC showed stronger tumor growth inhibition compared to its single-drug counterparts (412a-MMAF or 412a-SN38). DP-ADC development may mitigate the emergence of acquired resistance and potentially reduce the required dosage of each drug.

Several studies have explored DP-ADCs that incorporate one or more topoisomerase I inhibitors. While a majority of this research is preclinical, details regarding specific payload selection and conjugation methods remain undisclosed [22,23,24,25,26,27,28]. However, camptothecin analogs offer a compelling scaffold for linking two distinct drugs, leveraging their unique structure and ease of modification. These analogues have demonstrated advantages in linker site optimization, enhanced drug delivery efficiency, and improved conjugation stability. Consequently, bivalent ADCs that use camptothecin analogues show promise in overcoming tumor resistance and enhancing therapeutic efficacy, potentially representing a significant advancement in cancer treatment.

In addition to conventional ADC payloads, numerous unconventional options are being clinically explored to overcome drug resistance and broaden non-tumor activity. These include topoisomerase II inhibitors, RNA polymerase inhibitors, Bcl-xL inhibitors, STING agonists, KRAS inhibitors, and PARP inhibitors [55,56,57]. DP-ADCs represent a promising application for these payloads.

## 3. Challenges in the Analysis of DP-ADCs

### 3.1. Quantitative Analysis of the Payload and Payload-Linker

Several quantitative assays have been developed to measure payloads in biological samples. These include assays for SN-38, DXd, MMAE, MMAF, and exatecan [58,59]. While these methods are functional, further improvements in sensitivity and precision are needed to enhance lead compound screening and pharmacokinetic evaluations. Moreover, the development of methods capable of simultaneously quantifying multiple payloads and payload-linkers would be of immense value.

To optimize liquid chromatography–mass spectrometry (LC-MS) analyses for payloads and payload-linkers, the systematic optimization of mobile phase conditions and MS parameters is crucial [60]. This includes selecting an appropriate analytical column to define the mobile phase composition and gradient and adjusting the ionization mode, monitored ions, and voltage parameters to maximize detection [61,62,63]. A potential challenge that arises when using a mass spectrometer as a detector is that the in-source fragmentation of the payload-linker during MS analysis can hinder the detection of the free payload when using the selective reaction monitoring mode. This fragmentation is induced by ionization voltage and heat and can result in inaccurate quantifications. Microfluidic LC-MS is an emerging analytical technique that offers a promising alternative with the potential for increased sensitivity and reduced sample consumption [64].

Several quantitative analyses of the payload and payload-linker have been reported. For example, Olivier et al. presented an online solid-phase extraction-LC-tandem MS (online SPE-LC-MS/MS) method for quantifying the DM1 payload (a maytansinoid) in human serum that demonstrated high analytical performance and used only 0.25 mL of serum. The method provided a quantitative range of 0.200–200 ng/mL with an excellent precision of 0.9–4.4%, thereby validating its high sensitivity and specificity. It has been successfully applied to quantify serum DM1 concentrations following the intravenous administration of an investigational ADC carrying DM1 as its cytotoxic payload [65]. Wesley et al. developed a streamlined method that used HPLC-MS for rapid payload-linker quantitation. The method required only a low sample volume and demonstrated a lower limit of quantification of 10 nM. This analytical approach was successfully implemented to monitor the clearance of the payload-linker during the optimization of the tangential flow filtration production steps [60].

### 3.2. Quantitative Analysis of the Total Antibodies

**Figure 3:**
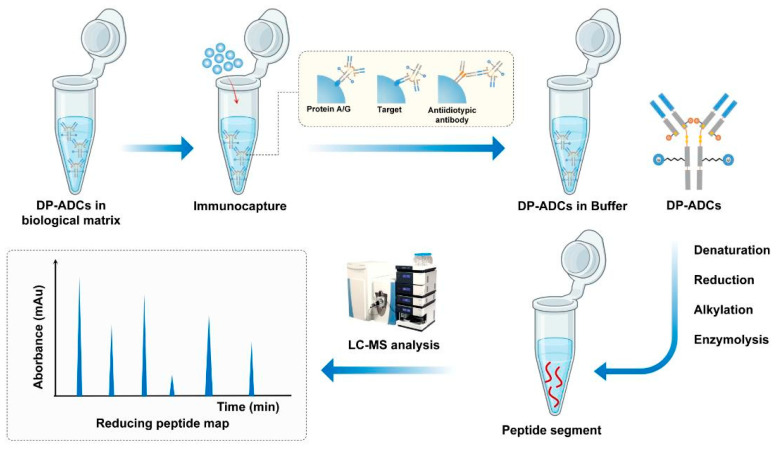
The workflow of pretreatment and LC-MS analysis of total antibody of dual-payloads antibody–drug conjugates (constructed and processed with Microsoft PowerPoint 2010).

The enzyme-linked immunosorbent assay (ELISA) and LC-MS are common quantitative methods for the analysis of total antibodies. ELISA is a mature and efficient technique that is suitable for routine batch testing, but it can be susceptible to interference from antibody cross-reactions. Researchers are increasingly using affinity LC-MS to address this interference, particularly in complex matrices, as it offers greater precision. During the quantitative analysis of total antibody by LC-MS, signature peptides are commonly used as practical surrogates for directly measuring the total antibodies [66], as shown in Figure 3. This approach is advantageous because intact antibodies ionize inefficiently, producing multiple charge states that hinder detection. The enzymatic digestion of antibodies into peptides circumvents this issue and significantly enhances analytical sensitivity [67]. Selecting an optimal surrogate peptide is therefore crucial and must follow specific criteria: the sequence must be unique to the target to avoid interference from endogenous proteins; it should originate from a stable, low-variability region of the antibody, such as the Fc domain; and the way the peptide’s amino acid composition affects the MS response must be considered. For example, selecting peptides that lack problematic residues, such as proline, can optimize the ionization efficiency and sensitivity [68,69].

Quantitative LC-MS analysis of ADCs requires using an appropriate internal standard to ensure precise and reproducible methods [70]. An ideal standard must remain chemically stable throughout the sample preparation, enzymatic digestion, and LC-MS analysis. Two primary surrogate types are employed: stable isotope-labeled (13C or 15N) ADC analogs and peptide sequence analogues [71]. Stable isotope-labeled ADCs are structurally identical to the target analyte. They provide the highest degree of compensation for variability across all analytical steps but are costly to synthesize. In contrast, peptide sequence analogues share similar amino acid sequences and are more affordable but primarily correct for LC-MS injection variability, offering more limited compensation for losses during extraction or digestion [72]. Therefore, selecting optimal internal standards requires a balanced consideration of the key sources of analytical variability, degree of molecular similarity required, and overall cost-effectiveness.

Accurate and reliable LC-MS quantification of ADCs using surrogate peptides necessitates the systematic optimization of both sample preparation and instrumental analysis [73]. The process begins with enzymatic digestion, where the selection of a suitable protease (e.g., trypsin, LysC, and Glu-C) is guided by the target peptide sequence. Additionally, the conditions, such as the enzyme-to-substrate ratio, temperature, pH, and digestion time, must be tightly controlled to minimize artifactual modifications [74]. Following this, solid-phase extraction is used for peptide purification and enrichment. The optimization of the sorbent and elution protocols is critical for peptide recovery and stabilization against processes like deamidation. For example, using a formic acid and acetonitrile solution in a hydrophilic–lipophilic balance reverse-phase mode can optimize this method. For the final LC-MS analysis, chromatographic separation is tailored to peptide properties. Reverse-phase columns are preferred for hydrophobic peptides, whereas hydrophilic interaction chromatography columns are used for hydrophilic variants. Further sensitivity gains are achieved by optimizing the mobile phase. This typically involves using acidic conditions to enhance electrospray ionization. In addition, stringent control of the solvent and additive purities is essential to suppress adduct formation and ensure high selectivity and sensitivity in detection [75,76].

Several LC-MS methods have been developed for the quantitative analysis of the total antibodies in biological matrices.

Yu et al. [77] developed a rapid and sensitive offline immuno-affinity LC-MS/MS method for the simultaneous quantification of total antibodies against DS001 and its free payload. This method utilizes a sample preparation workflow that involves immunocapture, denaturation, trypsin digestion, papain digestion, and reaction termination. When applied to rat serum, the method demonstrated good linearity between 125 and 2000 µg/mL, with a limit of quantification of 31.25 µg/mL.

In contrast to this offline method, Dan et al. [78] developed an online affinity LC-MS method that eliminated the need for sample pretreatment or enrichment. This method leverages the specific interaction between the column and total IgG-class antibodies coupled with high-resolution, high-accuracy MS for species identification. Consequently, the method allows for the rapid quantification of the total antibodies and the direct monitoring of key quality attributes, such as the dissociation ratio (DAR), in complex biological matrices. However, it is challenging to apply current online methods to determining the DAR of samples that contain dual payloads resulting from both sugar-coupling and thiol-coupling reactions due to limitations in online reduction processing capabilities.

Compared with single-payload ADCs (SP-ADCs), DP-ADCs pose greater quantitative analytical challenges due to their structural complexity and diverse components. SP-ADC analytical methods often benefit from established techniques that provide adequate separation, sensitivity, and specificity. However, DP-ADCs, with their increased payloads and complex degradation products, necessitate more sophisticated chromatographic separation. This may involve the use of advanced columns, optimization of gradient programs, or techniques such as two-dimensional liquid chromatography (2D-LC) or non-denaturing LC coupled with MS [78,79]. Similarly, traditional ELISAs may struggle to differentiate between multiple payloads, requiring the development of more specialized ELISAs that use a target protein specific to a single payload.

SP-ADCs are generally easier to handle analytically, and they often use acetonitrile or methanol for protein precipitation [80]. However, DP-ADCs, due to their complex composition, are prone to matrix effects that can reduce analytical accuracy. Consequently, these samples require more extensive purification and careful matrix matching. Effective sample processing involves isolation and enrichment of the target components while minimizing the interference. Strategies include exploring precipitation methods such as combining acetonitrile with acids or salts, and applying pre-treatment techniques such as affinity chromatography and solid-phase extraction [81]. Finally, variations in coupling methods and component stabilities across different payloads may induce degradation or transformation during sample processing, necessitating careful evaluation and control.

### 3.3. Determination of DAR and Its Distribution

It is well established that small-molecule payloads serve as a warhead enabling ADC-mediated tumor cell killing, while DAR represents the average number of payload molecules conjugated per antibody. Consequently, DAR is intrinsically linked to the antitumor activity of ADCs. However, a higher DAR can also lead to adverse outcomes. Given that most current small-molecule payloads possess hydrophobic structures, their conjugation imparts increased hydrophobicity to high-DAR ADCs. This elevated hydrophobicity predisposes these ADCs to aggregation, a major impediment in drug development. Additionally, it can accelerate systemic clearance due to enhanced immunogenicity and altered pharmacokinetic profiles. This ultimately limits in vivo exposure and efficacy [82]. Furthermore, the risk of off-target toxicity of high-DAR ADCs also escalates, which can limit the MTD of these ADCs. Seminal work by Hamblett et al. [83] demonstrated that for ADCs with the same design but different DAR values, the clearance rate of the ADC (DAR = 8) was three-fold faster than that of the ADC (DAR = 4) and five-fold faster than that of the ADC (DAR = 2). At equivalent antibody doses, the ADC (DAR = 4) exhibited superior in vivo antitumor activity compared with that of the ADC (DAR = 2). However, the antitumor activity of the ADC (DAR = 8) was only comparable with that of the ADC (DAR = 4). Moreover, the MTD of the ADC (DAR = 2) in mice was at least twice that of the ADC (DAR = 4) and four times that of the ADC (DAR = 8) [83]. These findings underscore that DAR is a critical quality attribute, warranting rigorous monitoring and control throughout ADC development and manufacturing. The scenario becomes more complex when transitioning from SP-ADCs to DP-ADCs, necessitating additional attention to the individual DAR of each payload. Significant differences in efficacy have been observed between DP-ADCs with the same total DAR but different DAR configurations. For example, Yamazaki et al. [13] reported that in the context of antitumor activity, a DP-ADC configured as MMAE/MMAF (4 + 2) demonstrated superior efficacy compared to an MMAE/MMAF (2 + 4) variant. From a bioanalytical perspective, payload-specific DAR is the most significant difference between DP-ADCs and their single-payload counterparts.

#### 3.3.1. Overview of Analytical Methods for Determination of DAR and Its Distribution

A variety of analytical methods are currently available for determining the DAR and its distribution in ADCs. UV/vis spectroscopy, liquid chromatography with ultraviolet detection (LC-UV), and LC-MS are the most commonly employed. UV/vis and LC-UV have been widely adopted for analyzing the DAR of FDA-approved ADCs [84], while LC-MS is increasingly utilized for characterizing novel ADC formats.

**Figure 4:**
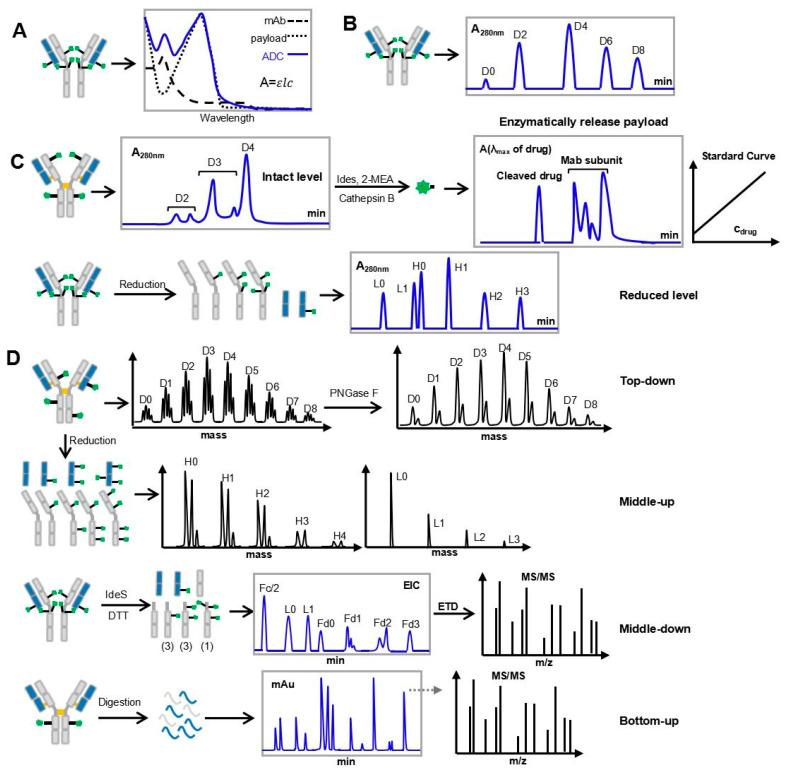
Schematic diagram of analytical methods for DAR and DAR distribution of ADCs. (**A**) A UV/vis spectroscopy method [85], (**B**) a HIC-UV method, (**C**) RPLC-UV methods based on intact level, reduced level or enzymatically released payload level [87], (**D**) LC-MS methods based on top-down, middle-up, middle-down [88] and bottom-up strategies. This figure was constructed and processed with Adobe Illustrator 29.8.4,and WPS Office Presentation 11.8.2.12195.

Relative to the other two analytical approaches, UV/vis spectroscopy offers notable advantages in terms of simplicity, speed, and user-friendliness [85]. It measures absorbance at the payload- and antibody-specific characteristic wavelengths, and the concentrations of both components can be determined according to the Lambert–Beer law, as shown in Figure 4A. The DAR value is subsequently calculated as the molar ratio of the payload concentration to the antibody concentration [86]. The prerequisite for DAR analysis using UV/vis spectroscopy is that the drug and the antibody possess distinguishable UV/vis characteristic absorption peaks, with their absorption behaviors exhibiting no mutual interference [86]. When spectral overlap occurs, absorbance at the characteristic wavelengths must be corrected to avoid overestimation of the DAR value [86]. Additionally, UV/vis spectroscopy is incapable of providing information on the DAR distribution, and the presence of free drugs in an ADC sample may lead to an overestimation of the calculated DAR [86].

LC-UV is currently the most commonly employed method for determining the DAR of FDA-approved ADCs due to its favorable accuracy and reproducibility [84]. Most of the payloads in current use possess pronounced hydrophobicity, and DAR species can be effectively resolved using hydrophobic interaction chromatography (HIC) or reversed-phase liquid chromatography (RPLC). The DAR and DAR distribution are subsequently derived from the peak area ratios of DAR species detected at 280 nm [84].

The HIC-UV method utilizes near-neutral pH-buffered salt solutions as the mobile phase, and under such conditions, ADCs can retain their native conformation [89]. This approach provides satisfactory separation for ADCs with limited heterogeneity, including cysteine-conjugated ADCs [90,91] and glycoengineered ADCs [92], as shown in Figure 4B. A distinct advantage of HIC-UV for DAR analysis lies in its capacity to directly furnish DAR distribution information from the chromatographic peak area ratios with a simple and rapid sample preparation workflow. In HIC-UV, the addition of high-concentration salt in the mobile phase may compromise peak reproducibility and resolution. The inclusion of isopropanol [93] or acetonitrile [94] may serve to improve the peak shape and separation efficiency, and concurrently enhance ADC recovery from the column [95]. Nevertheless, due to its limited separation efficiency, HIC-UV is not suitable for DAR analysis of highly heterogeneous ADCs, such as lysine-conjugated ADCs [89]. Recent methodological advancements in HIC-UV have primarily centered on optimizing elution conditions to achieve an enhanced resolution of DAR species [96,97].

In contrast to HIC-UV, RPLC-UV uses mobile phases that contain a high proportion of organic solvent and operate at elevated column temperatures. These conditions induce antibody denaturation. Consequently, cysteine-conjugated ADCs require reduction prior to analysis, and the DAR values are calculated from the relative chromatographic peak areas of the heavy chain and light chains [98], as shown in Figure 4C. For other site-specific conjugated ADCs, a direct analysis at the intact level is feasible [99], as shown in Figure 4C. RPLC-UV can provide a satisfactory resolution of DAR species across various conjugation formats. Increasing the column temperature has been shown to enhance the recovery of all ADC species from reversed-phase columns [100]. Additionally, the incorporation of ion-pairing agents into the mobile phase, typically 0.05% or 0.1% trifluoroacetic acid (TFA), helps mitigate peak broadening and the tailing of charged analytes [101,102]. However, RPLC-UV analysis is associated with certain limitations. Specific DAR species may be underrepresented due to column retention artifacts [89]. In addition, the reducing conditions used during sample preparation, together with the elevated temperatures and low-pH mobile phases used during separation, can promote the degradation of high-DAR ADC species. This may result in underestimating the DAR values [99,103,104]. Therefore, a careful balance must be struck between resolution and sample integrity. For ADCs with cleavable linkers, an alternative strategy is to enzymatically release the payload and quantify it using RPLC-UV. VC linkers can be cleaved using papain [105] or cathepsin B [87,106], while β-glucuronide linkers require β-glucuronidase [107]. To ensure complete cleavage, pretreatment with IdeS and a reducing agent is necessary to fully expose the conjugation sites, as shown in Figure 4C. While this method does avoid several limitations of the approaches discussed above, it fails to provide the DAR distribution. It also involves more complex sample preparation and requires an additional analysis to determine the antibody concentration for calculating the DAR value.

As the structural complexities of novel ADCs continue to increase, LC-MS is emerging as the most powerful approach for DAR characterization. On the chromatographic front, RPLC uses mobile phase conditions that are readily compatible with MS; however, caution is warranted regarding the potential for antibody degradation under elevated temperatures and a high-content organic solvent. Size-exclusion chromatography uses ammonium salt-based buffer systems, which also offer compatibility with MS, though the ionization efficiency provided by ammonium salts is considerably lower than that achieved with formic acid (FA) [108,109]. It has been reported that the inclusion of difluoroacetic acid during the mobile phase enhances MS sensitivity while providing superior chromatographic resolution compared with combinations of FA and TFA [110]. Conventional HIC, which relies on non-volatile mobile phases, is not directly amenable to MS; HIC-MS typically requires two-dimensional chromatography for desalting [111] or the use of specialized volatile mobile phases. Electrospray ionization (ESI) represents the most commonly used ion source for DAR analysis that uses MS. Quadrupole time-of-flight and Orbitrap are the most frequently used mass analyzers. Recent studies have also reported the application of triple quadrupole [112] and Fourier transform ion cyclotron resonance [113] for ADC characterization. Among these, Orbitrap offers the highest resolution and mass accuracy, albeit at the highest cost.

Compared with UV/vis and LC-UV, LC-MS is the only technique capable of identifying DAR species, providing information on conjugation sites, and determining site-specific occupancy. The accurate mass measurements provided by MS serve as robust evidence for the identification of DAR species, even when chromatographic separation is incomplete. Moreover, due to the high sensitivity of MS, LC-MS requires substantially less sample quantity than LC-UV. Four common strategies are used for the DAR determination by LC-MS: top-down, middle-up, middle-down, and bottom-up approaches [114], as shown in Figure 4D. The top-down method analyzes intact ADCs directly and is suited for ADCs with intact interchain disulfide bonds. This method provides direct information on the DAR distribution, as shown in Figure 4D. The middle-up approach analyzes ADCs following digestion with IdeS [73,115], reduction with dithiothreitol, or a combination thereof. This strategy offers subunit-level localization of payloads [116] while improving the mass resolution and sensitivity [86], as shown in Figure 4D. This method is particularly applicable to cysteine-conjugated ADCs. The middle-down method introduces ADC samples into the mass spectrometer, followed by fragmentation via electron transfer dissociation or electron transfer/high energy collision dissociation, as shown in Figure 4D. This approach enables the elucidation of conjugation sites without proteolytic digestion [116]. The bottom-up method analyzes peptides generated from ADC digestion and is the most effective strategy for identifying conjugation sites and determining site-specific occupancy [86], as shown in Figure 4D. However, because the payload may influence the proteolytic efficiency near conjugation sites, parallel or sequential digestion with two distinct enzymes is often necessary to ensure complete digestion. However, for lysine-conjugated ADCs, the digestion efficiency remains low even with such efforts [86]. PNGase F treatment can be used during sample preparation to remove N-glycans for ADCs, except N-glycan-conjugated ADCs, thereby reducing the complexity of deconvoluted mass spectra. However, the presence of hydrophobic payloads and/or hydrophobic linkers diminishes the ionization efficiency of DAR species in the ESI source [117]. In lysine-conjugated ADCs, this effect is further compounded by payload occupation of ionizable sites on the antibody. Collectively, these factors contribute to an underestimation of the DAR values measured by LC-MS. Recent studies have proposed strategies to address these limitations. One approach involves the enzymatic cleavage of the hydrophobic payload during sample preparation. This enables DAR analysis based on the residual linker [118]. Additionally, labeling unoccupied conjugation sites with linker-like structures has been shown to further minimize ionization efficiency disparities among different DAR species [119]. Alternatively, relative ionization efficiency factors can be applied to correct DAR values [117].

#### 3.3.2. DAR Analytical Challenges of DP-ADCs

Compared with SP-ADCs, DP-ADCs exhibit significantly greater diversity in DAR species, which imposes higher demands on method separation performance. During LC-UV analysis, native HIC-UV often fails to adequately resolve the DAR species of DP-ADCs, making it challenging to accurately determine the total DAR. Reduced RPLC-UV offers improved resolution over native HIC-UV, as reduction simplifies the DAR species profile. However, careful optimization of chromatographic conditions remains essential to achieve adequate separation. These optimizations include adding TFA and raising the column temperature. With these efforts, reduced RPLC-UV can provide information to determine the total DAR and indirectly estimate the total DAR distribution. However, because heavy chains with the same total DAR but different payload configurations often co-elute, particularly when the two payloads have similar hydrophobicity, reduced RPLC-UV generally cannot resolve individual DAR values for each payload. For DP-ADCs with cleavable linkers, enzymatic cleavage offers a route to obtain payload-specific DAR values. Payloads attached via cleavable linkers can be released simultaneously or sequentially using appropriate enzymes and quantified using RPLC-UV. Compared with SP-ADCs, sample preparation here may be more complex and potentially require two different linker-cleaving enzymes. Moreover, when the two payloads have similar hydrophobicity, chromatographic separation of the synchronously released payloads presents an additional challenge. For DP-ADCs that combine N-glycan conjugation with other conjugation chemistries, an alternative strategy involves the removal of N-glycan and its conjugated payload with PNGase F. This effectively converts the molecule into an SP-ADC, thereby enabling the DAR value and its distribution to be determined for the remaining conjugation site. The DAR value for the N-glycan site can then be derived by subtracting the known single-payload DAR from the total DAR. However, this strategy does not provide DAR distribution information for the N-glycan site. For other conjugation formats, enzymatic release of a single payload may leave linker remnants capable of altering the hydrophobicity of the ADC species. This can potentially complicate or even preclude DAR analysis for the remaining conjugation site using the approaches described above.

Compared with LC-UV, LC-MS provides more comprehensive information on DAR distribution of DP-ADCs. For SP-ADCs with intact interchain disulfide bonds, the DAR value and its distribution can be readily obtained using the top-down approach. However, for DP-ADCs, the top-down analysis suffers from a severe chromatographic peak overlap. The co-elution of DAR species that compete for ionization efficiency reduces the sensitivity and potentially masks signals from lower-abundance species. For site-specific payloads with high conjugation efficiencies, this often means only the most abundant DAR species are detectable. Therefore, for DP-ADCs with intact interchain disulfide bonds, top-down analysis is only suitable for assessing the total DAR value and its distribution, as well as the DAR value and distribution of non-site-specific payloads. Middle-up analysis effectively addresses this limitation. After reduction and/or digestion, the DP-ADC components are simplified. This leads to efficient peak separation and increased MS sensitivity. Simultaneously, as the m/z values of the analytes decrease, the mass accuracy improves, facilitating a reduced risk of misassignment. Thus, the middle-up method enables a more straightforward measurement of the DAR values for site-specific payloads and allows indirect assessments of the DAR distribution. Although MS generally offers a superior separation capability compared to LC, challenges arise when the two payloads have very similar masses. In such cases, ADC species with different DAR configurations produce closely spaced mass spectral peaks. This complicates deconvolution and increases the risk of misassignment, which places high demands on mass resolution. In this situation, Orbitrap can be particularly useful. Additionally, since DP-ADCs are often designed to achieve a high DAR, the negative impact of the payload hydrophobicity on the DAR measurement accuracy is particularly pronounced.

Overall, within the current technological landscape, determining the DAR and its distribution for DP-ADCs remains challenging, with difficulty largely dependent on the specific payload and linker types. A combination of orthogonal methods is often required for cross-validation, among which LC-MS demonstrates the greatest potential. Looking ahead, the continued development of novel ADC formats (e.g., bispecific DP-ADCs) and the application of new payload classes (e.g., hydrophilic small molecules) will inevitably introduce new complexities to DAR and DAR distribution analyses. Conversely, with continued advances in site-specific conjugation technologies, homogeneous ADCs are expected to become increasingly mainstream, which would substantially simplify the analysis of the DAR and its distribution.

## 4. Future Landscape

DP-ADCs represent a significant advancement in ADC technology and hold considerable promise for next-generation cancer therapy. However, their intrinsically complex molecular architectures and pharmacokinetic profiles present substantial challenges in terms of structural homogeneity, stability, and comprehensive characterization. Future development of DP-ADCs will focus on three key strategic areas.

### 4.1. Advanced Analytical Techniques for Rigorous Quality Control

Multi-tiered quality control strategies and advanced analytical methodologies are critical for characterizing these complex molecules and ensuring stringent product quality control. Key quality attributes related to the conjugation characteristics of DP-ADCs necessitate close monitoring. A multi-tiered quality control strategy may be necessary to consider establishing an encompassing detection and characterization at the intact, reduced subunit, and peptide mapping levels. Particular emphasis should be placed on critical parameters such as the average DAR, payload-specific DAR, conjugation site identification, and drug load distribution.

A technical perspective of conjugation characteristic analyses reveals that high-sensitivity and high-resolution analytical methods should be developed for the conjugation characterization. For example, online comprehensive 2D-LC enables the multi-dimensional profiling of conjugation attributes, real-time monitoring of the conjugation reaction process, and provides proactive data to support process optimization.

For free drug (and related) impurity (FDRI) analysis in DP-ADCs, the simultaneous presence of two payloads increases the risk of mutual interference. Therefore, separate detection methods for each payload are recommended, and individual qualification limits should be established based on the respective toxicity data. High-resolution MS in full-scan mode is a promising approach for FDRI testing. It allows for the simultaneous detection of multiple free payloads, degradation products, and cross-reaction byproducts, thereby enhancing the detection efficiency and analytical throughput. Additionally, total antibody quantification must ensure that the two payloads do not interfere with an accurate antibody determination to maintain the reliability of the bioanalytical measurements.

### 4.2. Innovative Payload Combinations and Rational Molecular Design

Therapeutic breakthroughs will depend on innovative payload combinations and rational molecular design. Beyond exploring novel cytotoxic agents, the integration of molecules with complementary mechanisms of action, such as immunomodulators (e.g., TLR agonists and STING agonists), RNA polymerase inhibitors, topoisomerase I inhibitors, and microtubule inhibitors, can generate synergistic DP-ADCs capable of overcoming tumor resistance. It is also advisable to employ linkers with distinct cleavage mechanisms to enable sequential or stimuli-responsive payload release to further enhance antitumor synergy and mitigate resistance.

Balancing the hydrophobicity of the two payloads is critical. For highly hydrophobic payloads, hydrophilic moieties that include polyethylene glycol, peptides (e.g., Gly-Gly-Phe-Gly), sugars (e.g., galactose), and sulfonic acid groups (e.g.,–SO3H) can be incorporated into linkers to reduce the overall hydrophobicity, prevent DP-ADC aggregation, and improve druggability. Furthermore, multimodal DP-ADCs designed as ternary or multivalent constructs that incorporate radioactive isotopes, photosensitizers, or immune agonists can achieve precise tumor targeting and multi-modal cancer cell killing, thereby enhancing therapeutic efficacy.

Site-specific conjugation technologies, particularly those that use non-natural amino acids at defined conjugation sites or enzymatic conjugation methods, have demonstrated strong potential to improve DAR control and increase the molecular homogeneity. The toxicity profiles of the different payloads should also be carefully considered. Ideally, payloads should exhibit non-overlapping toxicities to facilitate clinical management, reduce adverse effects, and improve patient compliance and treatment continuity.

### 4.3. High-Throughput Screening Platforms to Accelerate Development

High-throughput screening platforms will greatly accelerate DP-ADC research and development. Through the integration of advanced linker technologies, microfluidics, and machine learning algorithms driven by artificial intelligence, these platforms can efficiently evaluate diverse combinations of payloads, linkers, and antibodies to predict both the efficacy and safety profiles. These advancements will also shorten development timelines to facilitate clinical translation.

In summary, DP-ADCs are poised to become increasingly important tools for precision cancer therapy.

## Figures and Tables

**Table 1:** The critical quality control and corresponding analytical methods of ADCs [16,17].

Test Item	Instrumentation and Equipment
Mass	Liquid chromatography–mass spectrometry
Sequence coverage	
N-terminal sequencing	
C-terminal sequencing	
Post-translational modifications	
Conjugation sites confirmation and occupancy	
Secondary tertiary structure analysis	Circular dichroism spectroscopy
Differential scanning calorimetry	Differential scanning calorimeter
Peptide mapping analysis	Reversed-phase ultra-high-performance liquid chromatography
Charge variants analysis	Capillary isoelectric focusing electrophoresis/ion-exchange chromatography
Size variants analysis	Size-exclusion high-performance liquid chromatography
DAR value analysis	Reversed-phase high-performance liquid chromatography, hydrophobic interaction high-performance liquid chromatography, liquid chromatography–mass spectrometry
Unconjugated mAb	Hydrophobic interaction high-performance liquid chromatography
Free drug analysis	Reversed-phase high-performance liquid chromatography
Enzyme Residues	Enzyme-linked immunosorbent assay
Protein content analysis	Ultraviolet spectroscopy
Affinity analysis	Surface plasmon resonance, UNCLE, enzyme-linked immunosorbent assay, flow cytometer
Relative binding activity	Enzyme-linked immunosorbent assay
Biological activity	Cell base

**Table 2:** An overview of the reported dual-payload antibody–drug conjugate.

Payload	Linker	Linker-Conjugation	Antibody	DAR	Reference
MMAE and MMAF	MC-Cys(SiPr)-Cys(Acm)-PEG24	Maleimide	Anti-CD30	8 + 8	[18]
MMAE and MMAF	DBCO-PEG3-GluValCit-PABC-MMAE, TCO-PEG3-GluValCit-PABC-MMAF	MTGase-mediated transpeptidation	Anti-HER2, Anti-Trop2	4 + 2, 2 + 2, 2 + 4	[13]
MMAE and MMAF	MMAF-PAB-VC-NH-LacNAc oxazoline, MMAE-PAB-VC-NH-LacNAc oxazoline	Endo-S2-mediated glycosite-specific, thioester-based K248 site-specific	Anti-HER2	2 + 2	[19]
MMAE and DM1	MC-VcMMAE, SMCC-DM1	Maleimide and Lys residues	Anti-Tmab	2.83 + 5.25	[20]
MMAF and SN38	MV-PEG8-VC-PABC-SN38 and MC-PEG4-MMAF	Maleimide	Anti-EGFR, anti-cMET	2.4 + 1.7	[21]
PY-4CAR2 (TOP1i) and PE-E2K (microtubule inhibitor)	Undisclosed	Undisclosed	Anti-CDH6 and anti-FRα	Undisclosed	[22]
Antimetabolite inhibitor and TOP1i	Undisclosed	Undisclosed	Anti-HER2	Undisclosed	[23]
T01 (Alphatecan TOP1i) and MMAE	Undisclosed	Undisclosed	Anti-HER3 and anti-EGFR	4 + 2	[24]
TOP1i and EGFR TKI	Undisclosed	Enzymatic site-specific	Anti-HER3	Undisclosed	[25]
MMAE/dual TOP1i	Undisclosed	Undisclosed	Anti-Nectin-4	2 + 2 + 2	[26]
TOP1i and an immune agonist	Undisclosed	Undisclosed	Anti-Trop2	Undisclosed	[27]
Two different TOP1i	Undisclosed	Undisclosed	Anti-NaPi2b	2 + 2	[28]
Undisclosed	Undisclosed	Undisclosed	Anti-GPC3	Undisclosed	[31]
MMAE and novel payloads	Undisclosed	Undisclosed	Anti-HER2	Undisclosed	[32]
MMAF and IMQ	MC-C6-MMAF, phosphine-azide-IMQ	Maleimide and Lys residues	Anti-CD276/B7-H3	3–4 + 7–14	[33]
MMAE, Aldox, VBL, vcDox	MC-PEG3-VCP-payload MC-VCP-payload	Maleimide and cyclopentadiene	Anti-HER2	2 or 4 + 1.8–7.1	[34]
Tubulin and TOP1 inhibitors	Undisclosed	Undisclosed	Anti-Trop2	Undisclosed	[35]
Exatecan and MMAE	Undisclosed	Undisclosed	Anti-HER2	8 + 4	[36]
Topoisomerase I (Top1) inhibitor and a non-Top/non-Tubulin inhibitor	Undisclosed	Undisclosed	Anti-cMET/EGFR	4 + 4	[37]
DXd and MMAE	Undisclosed	Undisclosed	Anti-cMET	Undisclosed	[38]
Undisclosed	Undisclosed	Undisclosed	Anti-FRα	Undisclosed	[39]
MMAE and novel payloads	Undisclosed	Undisclosed	Anti-HER2	Undisclosed	[32]
Topoisomerase I inhibitor and a microtubule inhibitor	Undisclosed	Undisclosed	Anti-ITGB6/B7H3	Undisclosed	[40]

## Data Availability

No new data were created or analyzed in this study. Data sharing is not applicable to this article.

## Abbreviations

The following abbreviations are used in this manuscript:

DAR	drug-to-antibody ratio
DBCO	dibenzocyclooctyne
PEG	polyethylene glycol
GGFG	Gly-Gly-Phe-Gly
MMAF	monomethyl auristatin F
MMAE	monomethyl auristatin E
SN38	7-ethyl-10-hydroxycamptothecin
VC	Val-Cit
SMCC	succinimidyl4-(N-maleimidomethyl)cyclohexane-1-carboxylate
MTGase	microbial transglutaminase
PABC	p-aminobenzyloxycarbonyl
TCO	trans-cyclooctene
TOP1i	topoisomerase I inhibition
TKI	tyrosine kinase inhibitors
MC	maleimide
Aldox	doxorubicin
vcDOX	mc-vcp-doxorubicin
VBL	vinblastine
T-DA2	two cyclopentadiene residues
VCP	Va-Cit-p-aminobenzyloxycarbonyl
